# The Reducing Effects of Pyrogallol-Phloroglucinol-6,6-Bieckol on High-Fat Diet-Induced Pyroptosis in Endothelial and Vascular Smooth Muscle Cells of Mice Aortas

**DOI:** 10.3390/md18120648

**Published:** 2020-12-16

**Authors:** Seyeon Oh, Myeongjoo Son, Chul-Hyun Park, Ji Tae Jang, Kuk Hui Son, Kyunghee Byun

**Affiliations:** 1 Functional Cellular Networks Laboratory, Lee Gil Ya Cancer and Diabetes Institute, Gachon University, Incheon 21999, Korea; seyeon8965@gmail.com (S.O.); mjson@gachon.ac.kr (M.S.); 2 Department of Anatomy & Cell Biology, Graduate School of Medicine, Gachon University, Incheon 21936, Korea; 3 Department of Thoracic and Cardiovascular Surgery, Gachon University Gil Medical Center, Gachon University, Incheon 21565, Korea; cdgpch@gilhospital.com; 4 Aqua Green Technology Co., Ltd., Smart Bldg., Jeju Science Park, Cheomdan-ro, Jeju 63309, Korea; jtjang@aquagt.co.kr

**Keywords:** *ecklonia cava*, endothelial cells or vascular smooth muscle cells dysfunction, inflammasome, pyrogallol-phloroglucinol-6,6-bieckol, pyroptosis

## Abstract

In hyperlipidemia, pyroptosis in endothelial cells (ECs) induces atherosclerosis via the toll-like receptor 4 (TLR4) pathway. We evaluated the effects of Ecklonia cava extract (ECE) and pyrogallol-phloroglucinol-6,6-bieckol (PPB) on pyroptosis of ECs and vascular smooth muscle cells (VSMCs), which leads to attenuation of these cells and dysfunction of the aorta in high-fat-diet (HFD)-fed mice and in palmitate-treated ECs and VSMCs. The expression of TLR4 and nuclear factor kappa-light-chain-enhancer of activated B cells (NF-κB), which induce formation of NOD-LRR-and pyrin domain-containing protein 3 (NLRP3) inflammasomes, were increased by HFD and were decreased by ECE and PPB. The TLR4/NF-κB pathway was upregulated in palmitate-treated ECs and VSMCs and was decreased by ECE and PPB. The expressions of NLRP3/apoptosis-associated speck like protein containing a caspase recruitment domain, caspase-1, interleukin (IL)-1β, and IL-18 were increased by HFD and were decreased by ECE and PPB. Pyroptotic cells were increased by HFD and decreased by ECE and PPB. The expressions of the adhesion molecules, intercellular adhesion molecule and vascular cell adhesion molecule, and endothelin-1 were increased by HFD and were decreased by ECE and PPB. ECE and PPB decreased pyroptosis in the ECs and VSMCs, which was induced by HFD in the mouse aorta, and attenuated EC and VSMC dysfunction, an initiation factor of atherosclerosis.

## 1. Introduction

Atherosclerosis is an increasingly prevalent condition and induces cardiovascular diseases, including myocardial infarction, stroke, and peripheral arterial disease, which are leading causes of mortality worldwide [1]. Atherosclerosis is initiated from the activation or dysfunction of endothelial cells (ECs), followed by cell death and inflammation of the surrounding cells [2,3]. The death of ECs, vascular smooth muscle cells (VSMCs), macrophages, and other vascular cells is involved in the atherosclerosis process [4,5]. Activated or dying ECs secrete pro-inflammatory cytokines and adhesion molecules, such as P-selectin, intercellular adhesion molecule-1 (ICAM-1), and vascular cell adhesion molecule-1 (VCAM-1), which leads to migration and infiltration of monocytes and inflammatory cells [1,6]. Cytokines and adhesion molecules also increase the proliferation of VSMCs and induce the migration of these cells to the intimal surfaces of arteries, resulting in intimal hyperplasia, which hinders vasodilation [7].

Pyroptosis is a process of cell death and is involved in the formation of atherosclerotic regions [8]. Pyroptosis, as a pro-inflammatory and programmed cell death, leads to the rupture of cell plasma membranes and the release of pro-inflammatory mediators and cellular contents, which aggravate inflammation [9]. Pyroptosis is activated by various pathological conditions, which are known risk factors of atherosclerosis, including oxidative stress, hyperglycemia, dyslipidemia, and inflammation [10].

Under obese or dyslipidemia conditions, increased serum free fatty acids trigger inflammation by activating the toll-like receptor (TLR) pathway [11]. TLRs bind to endogenous metabolic danger-signal-associated molecular patterns (DAMPs) and induce upregulation of various inflammatory signaling pathways [12]. Additionally, TLRs induce pyroptosis by activating the transcription factor, NF-kB, which consequently upregulates the transcription of NOD-like receptor family pyrin domain-containing 3 (NLRP3) [13,14]. The complex form of the NLRP3 inflammasome is created by binding between activated NLRP3 and nucleate apoptosis-associated speck-like protein (ASC) containing a caspase activation and recruitment domain [15,16]. NLRP3 inflammasomes activate caspase-1 and converts inactive interleukin (IL)-β and IL-18 precursors to mature inflammatory cytokines. In addition, activated caspase-1 converts a member of the gasdermin D protein (GSDMD) to GSDMD-N, which facilitates the formation of membrane pores [13,14,15]. Membrane pores allow the release of inflammatory cytokines, such as activated IL-β and IL-18 [15,16], and induce cell swelling, due to the increased permeability of the plasma membrane, and water influx into cells, which eventually results in osmotic cell lysis [15,16]. Several studies have shown that caspase-1 promotes ECs activation and monocyte recruitment into the arterial intima in hyperlipidemia and lead to pyroptosis in ECs [5,17].

*Ecklonia cava,* (E. cava), an edible marine brown alga, shows anti-inflammatory and anti-obesity effects [18,19]. Pyrogallol-phloroglucinol-6,6-bieckol (PPB), a compound found in E. cava, significantly hinders monocyte migration and macrophage differentiation to the inflammatory type [20]; thus, PPB reduces monocyte-induced EC death and monocyte-induced VSMC proliferation and migration [20]. However, it has not yet been studied whether E. cava extract (ECE) or PPB can modulate the pyroptosis that is involved in EC and VSMC dysfunction. The purpose of the present study, therefore, is to evaluate the effects of ECE and PPB on the pyroptosis of ECs and VSMCs, which leads to the attenuation of these cells and cell dysfunction in the aorta of high-fat diet (HFD)-fed mice.

## 2. Results

### 2.1. ECE and PPB Reduced the Expression of TLR4 Increased by HFD in the Aorta and in Palmitate-Treated ECs and VSMCs

**Figure 1:**
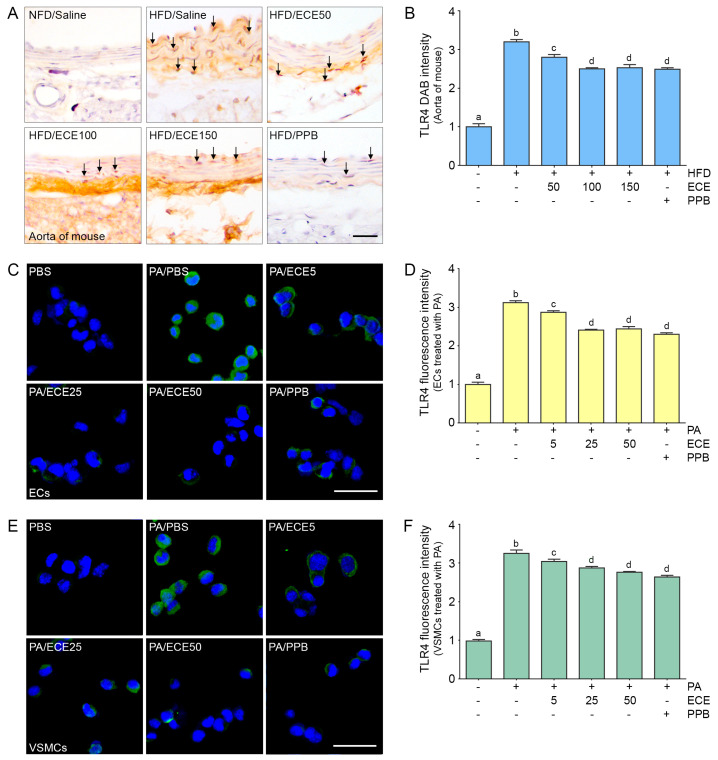
Reduction effects of E. cava extract (ECE) and pyrogallol-phloroglucinol-6,6-bieckol (PPB) on expression of toll-like receptor 4 (TLR4) in the aorta and in the palmitate treated endothelial cells (ECs) and vascular smooth muscle cells (VSMCs). (**A**,**B**) The protein expression of TLR4 in the aorta was increased by high-fat diet) HFD and was significantly decreased after treatment with ECE or PPB. Scale bar = 100 μm. (**C**–**F**) In ECs (SVEC4-10) and VSMCs (MOVAS), TLR4 protein levels were increased by palmitic acid (PA). Addition of ECE and PPB decreased the TLR4 expression levels. Scale bar = 200 μm. Data represent the means ± SD. Means identified to a different letter indicate significant differences between groups. DAB, 3,3-diaminobenzidine; ECs, endothelial cells; ECE, extract of *Ecklonia cava*; NFD, normal fat diet; HFD, high-fat diet; PA, palmitic acid; PBS, phosphate-buffered saline; PPB, pyrogallol-phloroglucinol-6,6-bieckol; SD, standard deviation; TLR4, toll like receptor 4; VSMCs, vascular smooth muscle cells.

The expression of TLR4 in the aorta of mice was increased by HFD and was subsequently decreased by administration of PPB or ECE as body weight or food consumption (Figure S1). The decreasing effect on TLR4 expression of 50 mg/mL of ECE was significantly lower than that of 100 and 150 mg/mL of ECE, however it was not significantly different between 100 and 150 mg/mL of ECE and PPB (Figure 1A,B).

The expression of TLR4 in mice ECs (Figure 1C,D) and VSMCs (Figure 1E,F) increased with palmitate treatment and was decreased by administration of PPB or ECE. The decreasing effect on TLR4 expression of 50 mg/mL of ECE was significantly lower than 100 and 150 mg/mL of ECE; however, it was not significantly different among 100 and 150 mg/mL of ECE and PPB.

### 2.2. ECE and PPB Reduced the Expression of NF-κB Increased by HFD in the Aorta and in Palmitate-Treated ECs and VSMCs

**Figure 2:**
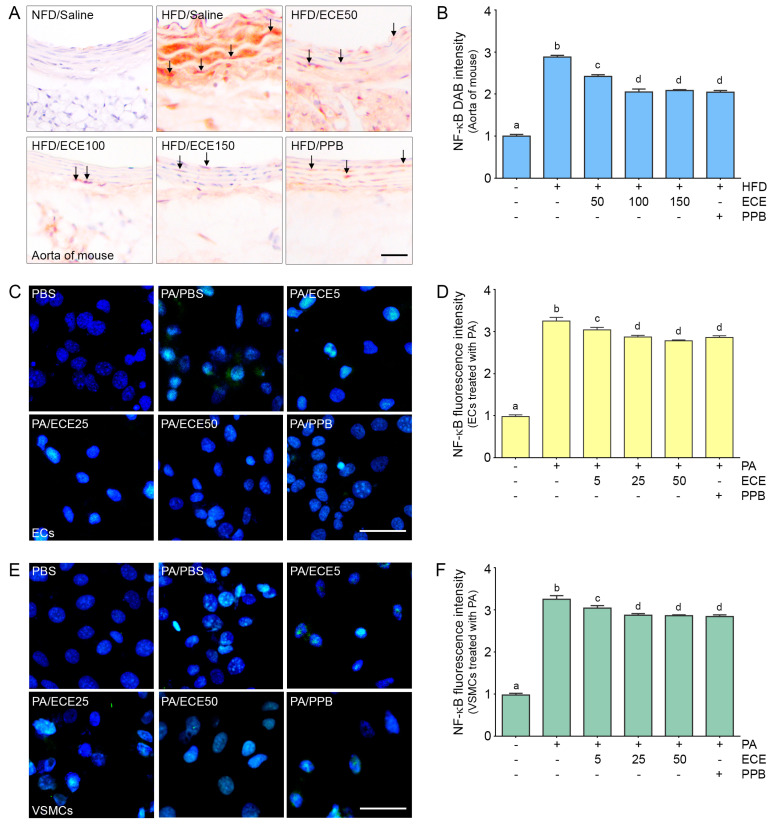
Decreased effects of ECE and PPB on expression of nuclear factor-kappa B (NF-κB) in the aorta and in the palmitate-treated ECs and VSMCs. (**A**,**B**) The protein expression of NF-κB in the aorta was increased by HFD and were significantly decreased after treatment with ECE or PPB. Scale bar = 100 μm. (**C**–**F**) In ECs (SVEC4-10) and VSMCs (MOVAS), NF-κB protein levels were increased by PA. Addition of ECE and PPB decreased the NF-κB expression levels. Scale bar = 200 μm. Data represent the means ± SD. Means identified with a different letter indicate significant differences between groups. DAB, 3,3-diaminobenzidine; ECs, endothelial cells; ECE, extract of *Ecklonia cava*; NFD, normal fat diet; NF-κB, nuclear factor-kappa B; HFD, high-fat diet; PA, palmitic acid; PBS, phosphate-buffered saline; PPB, pyrogallol-phloroglucinol-6,6-bieckol; SD, standard deviation; VSMC, vascular smooth muscle cells.

The expression of NF-κB in the aorta of mice was increased by HFD and was subsequently decreased by administration of PPB or ECE. The decreasing effect on NF-κB expression of 50 mg/mL of ECE was significantly lower than that of 100 and 150 mg/mL of ECE, however it was not significantly different between administration of 100 and 150 mg/mL of ECE and PPB (Figure 2A,B).

The expression of NF-κB in ECs (Figure 2C,D) and VSMCs (Figure 2E,F) in the mice was increased by palmitate treatment and decreased by PPB or ECE administration. The decreasing effect on NF-κB expression of 50 mg/mL of ECE was significantly lower than that of 100 and 150 mg/mL of ECE; however, it was not significantly different between administration of 100 and 150 mg/mL of ECE and PPB.

### 2.3. ECE and PPB Reduced the Expression of NLRP3 Increased by HFD in the Aorta and in the Palmitate-Treated ECs and VSMCs

**Figure 3:**
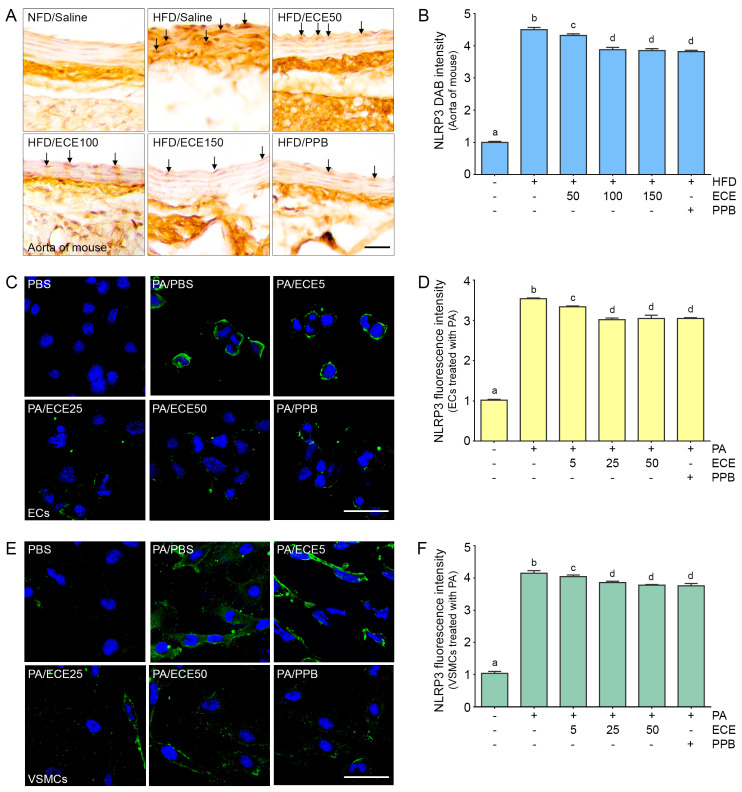
The reducing effects of ECE and PPB on expression of NOD-like receptor pyrin domain-containing protein 3 (NLRP3) in the aorta and in the palmitate-treated ECs and VSMCs. (**A**,**B**) The protein expression of NLRP3 in the aorta was increased by HFD and were significantly decreased after treatment with ECE or PPB. Scale bar = 100 μm. (**C**–**F**) In ECs (SVEC4-10) and VSMCs (MOVAS), NLRP3 protein levels were increased by PA. Addition of ECE and PPB decreased the NLRP3 expression levels. Scale bar = 200 μm. Data represent the means ± SD. Means identified with a different letter indicate significant differences between groups. DAB, 3,3-diaminobenzidine; ECs endothelial cells; ECE, extract of *Ecklonia cava*; NFD, normal fat diet; NLRP3, NOD-like receptor pyrin domain-containing protein 3; HFD, high-fat diet; PA, palmitic acid; PBS, phosphate-buffered saline; PPB, pyrogallol-phloroglucinol-6,6-bieckol; SD, standard deviation; VMCs, vascular smooth muscle cells.

The expression of NLRP3 in the aorta of HFD-fed mice was increased, and it was subsequently decreased by PPB or ECE. The decreasing effect on NLRP3 expression of 50 mg/mL of ECE was significantly lower than that of 100 and 150 mg/mL of ECE, however it was not significantly different between the administration of 100 and 150 mg/mL of ECE and PPB (Figure 3A,B).

The expression of NLRP3 in ECs (Figure 3C,D) and VSMCs (Figure 3E,F) was increased by treatment with PA and subsequently decreased by administration of PPB or ECE. The decreasing effect on NLRP3 expression of 50 mg/mL of ECE was significantly lower than that of 100 and 150 mg/mL of ECE; however, it was not significantly different between the administration of 100 and 150 mg/mL of ECE and PPB.

### 2.4. ECE and PPB Reduced the Expression of ASC, Which Was Increased by HFD in the Aorta and in Palmitate-Treated ECs and VSMCs

**Figure 4:**
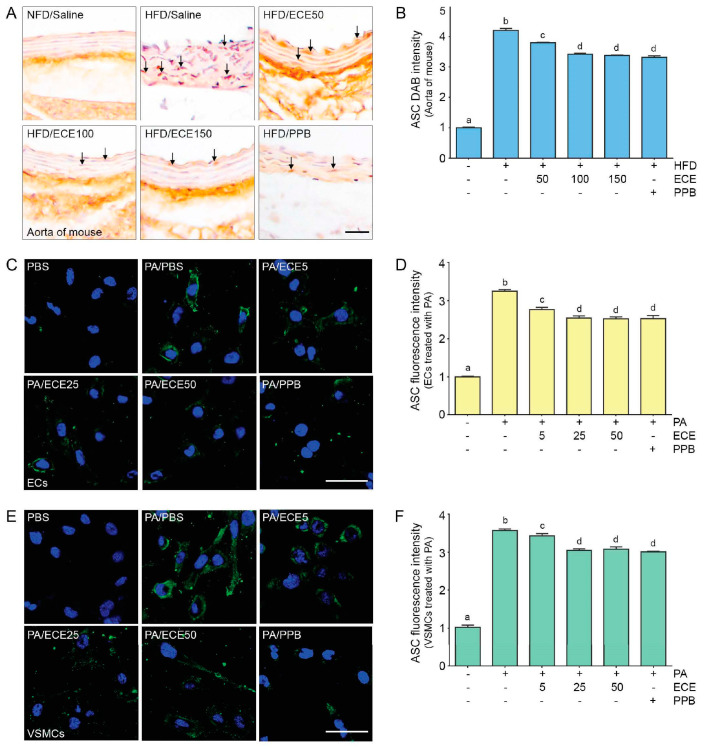
The reducing effects of ECE and PPB on expression of the adaptor molecule apoptosis-associated speck-like protein containing a CARD (ASC) in the aorta and in the palmitate-treated ECs and VSMCs. (**A**,**B**) The protein expression of ASC in the aorta was increased by HFD and were significantly decreased after treatment with ECE or PPB. Scale bar = 100 μm. (**C**–**F**) In EC (SVEC4-10) and SMC (MOVAS), ASC protein levels were increased by PA. Addition of ECE and PPB decreased the ASC expression levels. Scale bar = 200 μm. Data represent the means ± SD. Means identified with a different letter indicate significant differences between groups. ASC, the adaptor molecule apoptosis-associated speck-like protein containing a CARD; DAB, 3,3-diaminobenzidine; ECs, endothelial cells; ECE, extract of *Ecklonia cava*; NFD, normal fat diet; HFD, high-fat diet; PA, palmitic acid; PBS, phosphate-buffered saline; PPB, pyrogallol-phloroglucinol-6,6-bieckol; SD, standard deviation; VSMCs, vascular smooth muscle cells.

The expression of ASC in the aortas of mice was increased by the HFD and was subsequently decreased by administration of PPB or ECE. The decreasing effect on ASC expression of 50 mg/mL of ECE was significantly lower than that of 100 and 150 mg/mL of ECE, however it was not significantly different between the administration of 100 and 150 mg/mL of ECE and PPB (Figure 4A,B).

The expression of ASC in ECs (Figure 4C,D) and VSMCs (Figure 4E,F) was increased by treatment with palmitate acid and decreased by the administration of PPB or ECE. The decreasing effect on ASC expression of 50 mg/mL of ECE was significantly lower than that of 100 and 150 mg/mL of ECE; however, it was not significantly different between the administration of 100 and 150 mg/mL of ECE and PPB.

### 2.5. ECE and PPB Reduced Expressions of Caspase-1, IL-1β, and IL-18, Which Were Increased by HFD in the Aorta and in Palmitate-Treated ECs and VSMCs

**Figure 5:**
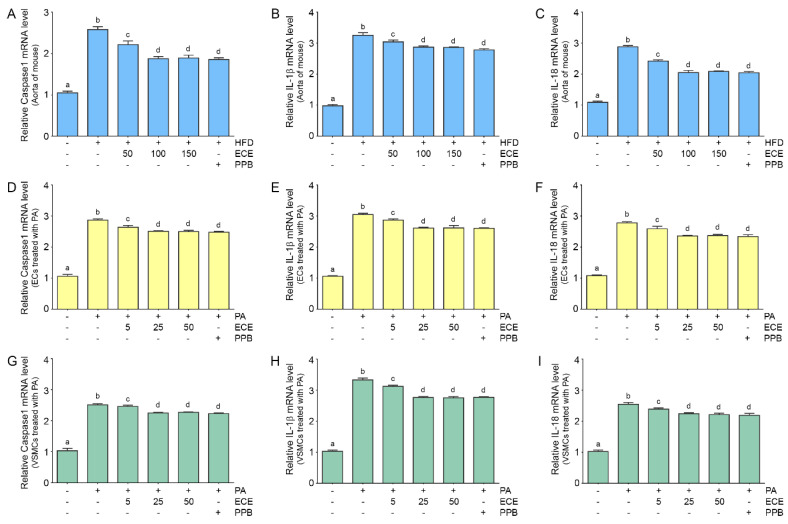
The reduction effects of ECE and PPB on expression of Caspase1, Il-1β and IL-18 in the aorta and in the palmitate-treated ECs and VSMCs. (**A**–**C**) The mRNA expression of Caspase1, Il-1β and IL-18 in the aorta was increased by HFD and were significantly decreased after treatment with ECE or PPB. (**D**–**I**) In ECs ((**D**–**F**), SVEC4-10) and VSMCs ((**G**–**I**), MOVAS), Caspase1, Il-1β and IL-18 mRNA levels were increased by PA. Addition of ECE and PPB decreased the Caspase1, Il-1β and IL-18 expression levels. Data represent the means ± SD. Means identified with a different letter indicate significant differences between groups. ECs, endothelial cells; ECE, extract of *Ecklonia cava*; HFD, high-fat diet; IL-1β, interleukin-1 beta; IL-18, interlurkin-18; PA, palmitic acid; PBS, phosphate-buffered saline; PPB, pyrogallol-phloroglucinol-6, 6-bieckol; SD, standard deviation; VSMCs, vascular smooth muscle cells.

The expressions of caspase-1, IL-1β, and IL-18 in the mouse aortas were increased by the HFD and were subsequently decreased by administration of PPB or ECE. The decreasing effect on the expressions of caspase-1, IL-1β, and IL-18 of 50 mg/mL of ECE was significantly lower than that of administration of 100 and 150 mg/mL of ECE; however, they were not significantly different between administration of 100 and 150 mg/mL of ECE and PPB (Figure 5A–C).

The expressions of caspase-1, IL-1β, and IL-18 in ECs (Figure 5D–F) and VSMCs (Figure 5G–I) were increased by treatment with palmitate and decreased by administration of PPB or ECE. The decreasing effects on caspase-1, IL-1β, and IL-18 expression of 50 mg/mL of ECE were significantly lower than that of 100 and 150 mg/mL of ECE; however, they were not significantly different between administration of 100 and 150 mg/mL of ECE and PPB.

### 2.6. ECE and PPB Reduced Pyroptosis, Which Was Increased by HFD, in the Aorta and in Palmitate-Treated ECs and VSMCs

**Figure 6:**
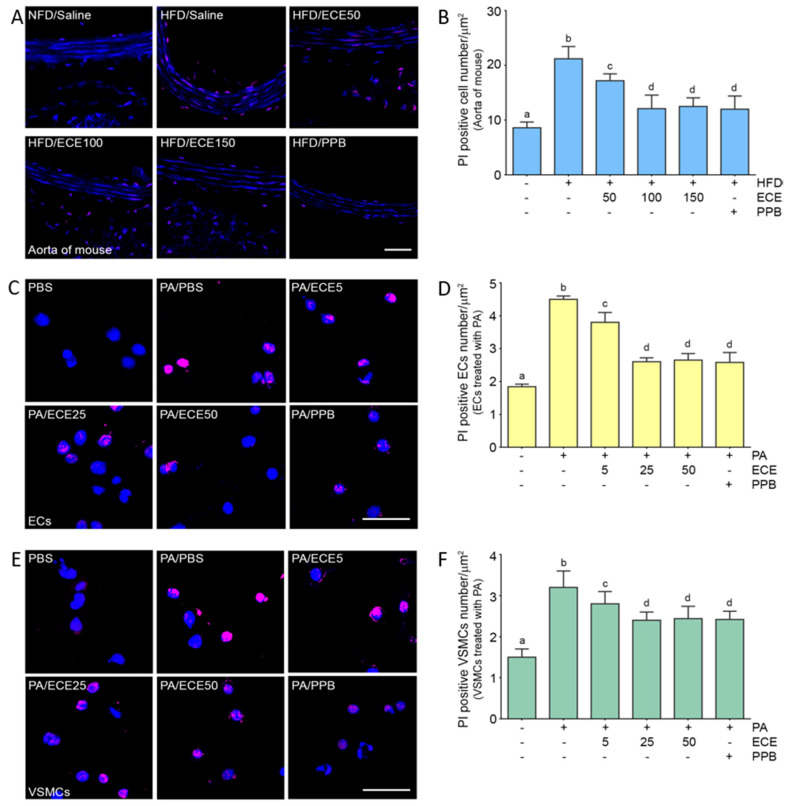
The reducing effects of ECE and PPB on pyroptosis in the aorta and in the palmitate-treated ECs and VSMCs. (**A**,**B**) The PI positive cell number in the aorta was increased by HFD and was significantly decreased after treatment with ECE or PPB. Scale bar = 100 μm. (**C**–**F**) In ECs (SVEC4-10) and VSMCs (MOVAS), PI positive cell number were increased by PA. Addition of ECE and PPB decreased PI positive cell number. Scale bar = 200 μm.Data represent the means ± SD. Means identified with a different letter indicate significant differences between groups. ECs, endothelial cells; ECE, extract of *Ecklonia cava*; NFD, normal fat diet; HFD, high-fat diet; PA, palmitic acid; PBS, phosphate-buffered saline; PI, propidium iodide staining; PPB, pyrogallol-phloroglucinol-6,6-bieckol; SD, standard deviation; VSMC, vascular smooth muscle cells.

Pyroptotic cells were positively stained by propidium iodide (PI) [21]. The number of positively stained cells in the aorta of mice was increased by the HFD and were subsequently decreased by administration of PPB or ECE (Figure 6A,B). The decreasing effect on the number of pyroptotic cells by administration of 50 mg/mL of ECE was significantly lower than that of 100 and 150 mg/mL of ECE; however, it was not significantly different between administration of 100 and 150 mg/mL of ECE and PPB.

The number of pyroptotic cells in ECs (Figure 6C,D) and VSMCs (Figure 6E,F) was increased by treatment with palmitate acid and decreased by administration of PPB or ECE. The decreasing effect on pyroptotic cells of 50 mg/mL of ECE was significantly lower than that of 100 and 150 mg/mL of ECE; however, it was not significantly different between administration of 100 and 150 mg/mL of ECE and PPB. 

### 2.7. ECE and PPB Reduced Cell Dysfunction in the Aorta and in Palmitate-Treated ECs and VSMCs

**Figure 7:**
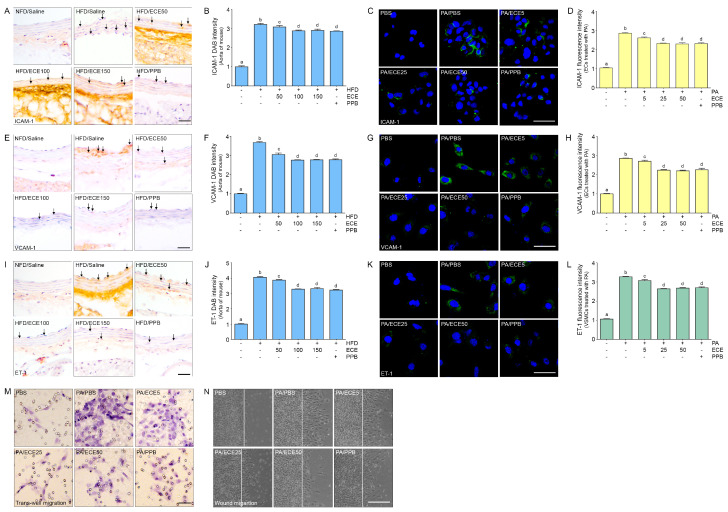
The improving effects of ECE and PPB on vascular functions in the aorta and in the palmitate-treated ECs and VSMCs. (**A**,**B**) The ICAM-1 expression in the aorta was increased by HFD and was significantly decreased after treatment with ECE or PPB. Scale bar = 100 μm. (**C**,**D**) In ECs (SVEC4-10), ICAM-1 expression increased by PA. Addition of ECE and PPB decreased ICAM-1 expression. Scale bar = 200 μm. (**E**,**F**) The VCAM-1 expression in the aorta was increased by HFD and were significantly decreased after treatment with ECE or PPB. Scale bar = 100 μm. (**G**,**H**) In ECs (SVEC4-10), VCAM-1 expression increased by PA. Addition of ECE and PPB decreased VCAM-1 expression level. Scale bar = 200 μm. (**I**,**J**) The ET-1 expression in the aorta was increased by HFD and was significantly decreased after treatment with ECE or PPB. Scale bar = 100 μm. (**K**,**L**) In VSMCs (MOVAS), ET-1 expression increased by PA. Addition of ECE and PPB decreased ET-1 expression level. Scale bar = 200 μm. (**M**,**N**) In VSMCs (MOVAS), trans-well migration and wound migration cell number increased by PA. Addition of ECE and PPB decreased. Scale bar = 100 μm.Data represent the means ± SD. Means identified with a different letter indicate significant differences between groups. ECs, endothelial cells; ECE, extract of *Ecklonia cava*; ET-1, endothelin-1; NFD, normal fat diet; HFD, high-fat diet; ICAM-1, intercellular adhesion molecule-1; PA, palmitic acid; PBS, phosphate-buffered saline; PI, propidium iodide staining; PPB, pyrogallol-phloroglucinol-6,6-bieckol; SD, standard deviation; VCAM-1, vascular cell adhesion molecule-1; VSMCs, vascular smooth muscle cells.

The expressions of the adhesion molecules, ICAM-1 and VCAM-1, and endothelin-1 (ET-1) in the aorta of mice were increased by the HFD and were decreased by administration of PPB or ECE (Figure 7A,B,E,F,I,J). The decreasing effect on the expressions of ICAM-1 and VCAM-1 by the administration of 50 mg/mL of ECE was significantly lower than that of 100 and 150 mg/mL of ECE; however, it was not significantly different between the administration of 100 and 150 mg/mL of ECE and PPB. The expressions of ICVAM and VCAM in SVEC4-10 were increased by treatment with palmitate and decreased by administration of PPB or ECE (Figure 7C,D,G,H). The expression of ET-1 in MOVAS was increased by treatment with palmitate and decreased by administration of PPB or ECE (Figure 7K,L). The trans-well migration and wound migration cell number in MOVAS was increased by treatment with PA and decreased by administration of PPB or ECE (Figure 7M,N).

## 3. Discussion

Under dyslipidemic and inflammatory conditions such as hyperlipidemia, hypercholesterolemia, or obesity, caspase-1 activation leads to the upregulation of the inflammasome pathway, activated by elevated lipids or inflammatory mediators, such as DAMPs, in ECs [5,12]. Activated caspase-1 promotes pyroptosis, which results in plasma membrane rupture and the release of inflammatory factors [22]. DAMPs and free fatty acids activate TLRs [12] and lead to the upregulation of NF-κB, which results in increasing NLRP3 inflammasomes [13,14].

In our study, expression of the TLR4/NF-κB pathway was increased in the aorta of HFD-fed mice and was decreased by administration of either ECE or PPB. The expression of NLRP3 inflammasomes, which was evaluated by the expression of both NLRP3 and ASC, was increased in the aorta of HFD-fed mice and was also decreased by the administration of ECE or PPB.

Oxidized low-density lipoprotein, palmitate, and saturated fatty acids have been previously reported to promote pyroptosis in ECs [23].

In our study, we made an in vitro hyperlipidemia model by treating ECs and VSMCs with PA. The expression of the TLR4/NF-κB pathway was increased by treatment with palmitate in ECs as well as VSMCs. NLRP3 inflammasomes were also increased in ECs and VSMCs by treatment with palmitate.

Hyperlipidemia results in the upregulation of NLRP3, caspase-1, and IL-1β, and leads to pyroptosis in ECs [5]. Similar with previous reports, our study showed that expression of caspase-1, IL-1β, and IL-18 in the aorta of HFD-fed mice was increased and was decreased by administration of either ECE or PPB. The level of pyroptosis, evaluated by PI staining, was increased in the aorta of HFD-fed mice and was decreased after administration of either ECE or PPB. Expressions of caspase-1, IL-1β, and IL-18, and the level of pyroptosis, were increased in ECs and VSMCs by treatment with palmitate.

After increased EC death, the reduced number and integrity of the ECs results in increased permeability of the intimal ECs monolayer [5]. This increased permeability leads to the migration and deposition of lipids, monocytes, and VSMCs into the intimal layer and aggravates further damage [5]. Injured ECs in the monolayer encourages the migration of VSMCs to the injured site, increases VSMC proliferation, and causes intimal hyperplasia [5]. A complete EC monolayer is necessary to regulate VSMC function [24]. EC death leads to a disrupted vasomotor response and causes reduced vasodilation and increased contraction. EC death also increases vascular inflammation via promotion of vasoconstrictor agents and adhesion molecules [4,25]. Early lesions of atherosclerosis are characterized by vasodilatory dysfunction and ECs [26,27]. Many studies have shown that pyroptosis of EC is involved in atherosclerosis, especially in early atherosclerotic vascular injuries [5,17,28]. Activated endothelial cells at sites of early atherosclerosis have been reported to show increased expression of P-selectin, ICAM-1, and VCAM-1. It has also been reported that recruitment of monocytes into the intima is increased after HFD feeding of apolipoprotein E −/− mice [28], and that caspase-1 is increased in ECs, accompanied by increasing expression of ICAM-1 and VCAM-1 [5,17]. The previous studies suggested that caspase-1 activation by hyperlipidemia leads to EC activation, caused pyroptosis of ECs, and increased ECs adhesion molecule production, which increases the adhesion of ECs to monocytes [5,17].

In our study, after mice were fed on HFD, the expressions of ICAM-1 and VCAM-1 were increased in the aorta, and these expression levels were decreased by administration of either ECE or PPB. The expressions of ICAM-1 and VCAM-1 in the ECs that were treated with palmitate were increased and were subsequently decreased by administration of either ECE or PPB. It seems that ECE and PPB can decrease the expression of EC adhesion molecules by decreasing pyroptosis.

During atherosclerosis progression, the increase in the expression of endothelin-1 (ET-1) is accompanied by proliferation [29], migration [30], and contraction of VSMCs [31], which are involved in matrix remodeling [32,33] and synthesis of the extracellular matrix [34]. Many studies have reported that the expression of ET-1 and its receptors is upregulated in both atherosclerosis animal models [35,36] and human atherosclerotic lesions [37,38,39]. ET-1 is also involved in intimal hyperplasia formation by increasing VSMC proliferation and migration [40]. In our study, expression of endothelin-1 was increased in the aorta of HFD-fed mice and it was decreased by administration of ECE or PPB. The expression of endothelin-1 was also increased in VSMCs treated with palmitate and was decreased by ECE or PPB.

In our study, we did not use TLR4 knock-out mice or inhibition study for TLR4 by using siRNA to evaluate whether the TLR4 signal pathway definitively initiated pyroptosis. Thus, we may not definitively say that ECE or PPB decreased pyroptosis through the TLR4 pathway. In the future study, it should be evaluated whether ECE or PPB directly decreased pyroptosis through the TLR4 pathway with TLR4 knock-out mice.

In conclusion, our results show that ECE and PPB decreased the expressions of the TLR4 and NF-κB pathways, and the expressions of caspase-1, IL-1β, and IL-18, and decrease pyroptosis which was increased in the aorta. The results of this study also show that adhesion molecules of ECs and ET-1, molecules which are known to be involved in atherosclerosis formation, are decreased by ECE and PPB.

## 4. Materials and Methods

### 4.1. Ecklonia Cava Extract (ECE) and Pyrogallol-Phloroglucinol-6,6-Bieckol (PPB) Preparation

ECE and PPB preparation methods were followed from previous study [41].

### 4.2. Diet Induced Obesity Animal Model

Male C57BL/6N mice (8 weeks of age) were obtained from Orient Bio (Seongnam, Korea) and kept at a constant temperature of 23 °C, relative humidity of 50%, and a dark/light cycle of 12/12 hrs. Mice were fed different diets as described below and provided drinking water ad libitum for eight weeks. For the first four weeks, mice received either a regular normal fat diet (NFD), or a 45% high fat diet (HFD; Research diet, New BrunswickNJ, USA) adapted from a previous study [42].

For the last four weeks, HFD mice were orally administered 0.9% normal saline, ECE (HFD/ECE; 50, 100, 150 mg/kg/day) or PPB (HFD/PPB; 2.5 mg/kg/day), along with either an NFD or HFD [43]. At the end of the eight-week study period, all mice were euthanized in accordance with the ethical principles in institutional animal care and use committee of Gachon university (approval number; LCDI-2019-0097).

### 4.3. Cell Culture and Experimental Cell Models

Vascular endothelial cells (SVEC4-10) and vascular smooth muscle cells (MOVAS) were purchased from the American Type Culture Collection and cultured with high-glucose dulbecco’s modified eagle medium (Hyclone, Marlborough, MA, USA) 10% fetal bovine serum (Millipore, Danvers, MA, USA), and penicillin/streptomycin (Gibco, Gaithersburg, MD, USA) at 37 °C under 5% CO_2_. Vascular smooth muscle cells were treated with 200 μm palmitate acid for 24 h based on previous studies [20]. As the same time, ECs and VSMCs were treated with ECE (5, 25, and 50 µg/mL) and PPB (1.8 µg/mL) for 24 h based on previous studies [20,43].

### 4.4. RNA Extraction and cDNA Synthesis

Total RNA was extracted using RNAiso Plus (Takara, Kyoto, Japan) according to the manufacturer’s instructions [44]. RNAiso Plus (0.5 mL) was mixed with chloroform (0.1 mL) and incubated at room temperature for 7 min. The mixture was centrifuged for 15 min at 4 °C at 12,000× *g*. The supernatant was collected in a new tube, mixed with 0.25 mL of 100% isopropanol, gently shaken, and then centrifuged again to precipitate the RNA. The supernatant was discarded, and the RNA pellet was washed with 70% ethanol and centrifuged at 7500× *g* for 5 min at 4 °C. The dried pellet was dissolved in 30 μL of diethyl pyrocarbonate water and the RNA was quantified using a NanoDrop 2000 spectrophotometer. Total RNA (1 µg) was used to synthesize cDNA using a cDNA synthesis kit (Takara, Kyoto, Japan).

### 4.5. Real-Time Reverse Transcription Polymerase Chain Reaction (qRT-PCR)

qRT-PCR was performed to analyze the mRNA levels. The reaction mixtures were prepared in 384-well plates and contained 0.8 µL of 10 pM primer (Table S1), 1 µg cDNA template (2 µL), and 5 µL SYBR Green (Takara, Kyoto, Japan). Analysis was performed with a CFX384 Touch Real-Time PCR Detection System (Bio-Rad, Hercules, CA, USA).

### 4.6. Immunohistochemistry (3,3-diaminobenzidine; DAB)

Blocks of paraffin-embedded aorta tissue were sectioned to a thickness of 7 µm, placed on a coating slide and dried at 40 °C for 24 h. Slides were then deparaffinized and incubated in 0.3% H_2_O_2_ (Sigma-aldrich, Missouri, MO, USA) for 30 min. Slides were subsequently rinsed three times with PBS and incubated in normal animal serum to block non-specific binding and incubated with primary antibodies (Table S2) at 4 °C, followed by three additional rinses with PBS. Slides were then treated with biotinylated secondary antibodies from the ABC kit (dilution rate 1:200; Vector Laboratories, Burlingame, CA, USA), incubated for 1 h with blocking solution, and rinsed three times with PBS. Slides were left to react with DAB substrate for 15 min, followed by mounting with a cover slip and dibutylphthalate polystyrene xylene (Sigma-aldrich, Missourti, MO, USA) mounting solution. Images were detected using a light microscope (Olympus, Tokyo, Japan) and quantification of the intensity of the brown color was performed using Image J software (NIH, Maryland, MD, USA) [45,46].

### 4.7. Immunocytochemistry

The cells were seeded at 3 × 10^5^ cells/well in 8-well chambers and treated with PA, ECE5, 25, 50μg/mL and PPB. the slides were fixed in ice-cold methanol for 10 min. After fixation, the slides were washed in PBS and treated with normal serum for 1 h at room temperature to reduce nonspecific antigen-antibody interaction. Primary antibodies were incubated for 24 h, and then secondary antibodies were incubated for 1 h at room temperature (Table S2). After washing cells with PBS, the samples were treated with 4′6-diamino-2-phenilindole (DAPI, Sigma Aldrich, D3286) for 3 min at room temperature to stain nuclei. The slides were mounted and photographed with a confocal microscope (Zeiss, LSM 710) and images were analyzed with Zen 2009 software (Zeiss).

### 4.8. Trans-Well Migration Assay

VSMCs were seeded at a density of 5 × 10^4^ per well onto 8 µm Transwell inserts (Thermo Fisher Scientific, Waltham, MA, USA). The lower chamber was filled with 500 μL low serum medium containing ECE5, 25, 50 μg/mL or PPB and PA and incubated for 48 h at 5% CO_2_ incubator. Migration activities were evaluated using water-soluble tetrazolium salts (WST; Daeil Lab Service, Republic of Korea) and optical densities were measured.

### 4.9. Wound Migration Assay

VSMCs (1 × 10^4^ cells per well) were seeded into a 12-well plate and cultured to confluence (2 days) and then FBS-starved for 24 h. One perpendicular scratch was then made in each well using a 1 ml pipette tip, and the medium was replaced with medium without FBS or starvation medium containing ECE5, 25, 50 μg/mL or PPB and PA. Images were taken 16 h later using an Axio Observer apparatus and analyzed using Wimasis image analysis software.4.10. 

The Kruskal–Wallis test and the Mann–Whitney U as a post-hoc test were used to determine the significance of differences among groups treated with palmitate only and groups treated with palmitate and ECE or PPB. Experiments were performed in triplicate, and results are presented as means ± SD. The analyses were conducted using SPSS version 22 (IBM Co., Armonk, NY, USA).

## Supplementary Materials

The following are available online at https://www.mdpi.com/1660-3397/18/12/648/s1, Table S1: List of primer for qRT-PCR; Table S2: List of antibodies for immunohistochemistry and immunocytochemistry; Figure S1: The reducing effects of ECE and PPB on body weight and food consuption in HFD -fed mice; Figure S2: The reducing effects of ECE and PPB on expression of NLRP3 and ASC in the aorta and in the palmitate treated ECs and VSMCs.
