# Supplementary material for: The Reducing Effects of Pyrogallol-Phloroglucinol-6,6-Bieckol on High-Fat Diet-Induced Pyroptosis in Endothelial and Vascular Smooth Muscle Cells of Mice Aortas
Source: Mar Drugs. 2020 Dec 16;18(12):648. doi: 10.3390/md18120648 (PMC7766911; doi:10.3390/md18120648)
Supplement: Supplementary file 1 [file marinedrugs-18-00648-s001.pdf]

The reducing effects of pyrogallol-phloroglucinol-6,6-bieckol on high-fat diet-induced pyroptosis in endothelial and vascular smooth muscle cells of mice aortas

Seyeon Oh[1,#], Myeongjoo Son[1,2], Chul-Hyun Park[3], Ji Tae Jang[4], Kuk Hui Son[3,*], Kyunghee Byun[1,2,*]

[1] Functional Cellular Networks Laboratory, College of Medicine, Department of Medicine, Graduate School and Lee Gil Ya Cancer and Diabetes Institute, Gachon University, Incheon 21999, Republic of Korea (S.O.; seyeon8965@gmail.com)

[2] Department of Anatomy & Cell Biology, Gachon University College of Medicine, Incheon 21936, Republic of Korea (M.S.; mjson@gachon.ac.kr)

[3] Department of Thoracic and Cardiovascular Surgery, Gachon University Gil Medical Center, Gachon University, Incheon 21565, Republic of Korea

[4] Aqua Green Technology Co., Ltd, Smart Bldg., Jeju Science Park, Cheomdan-ro, Jeju 63309, Republic of Korea

* Correspondence: Kuk Hui Son (dr632@gilhospital.com), Tel: +82-32-460-3666; Kyunghee Byun (khbyun1@gachon.ac.kr), Tel: +82-32-899-6511

# These authors contributed equally to this work.

Supplementary Tables

Supplementary table 1. List of primer for qRT-PCR

Gene		Primers
actb	Forward	5'-ACA AAG CTG TTC AGT GTC TCC A-3’
	Reverse	5'-CTC CGT TTC CAG AAT ACA CAC A-3’
Caspase1	Forward	5'- AGG ACA TCC TTC ATC CTC AGA A-3’
	Reverse	5'- TTC TAA AGG GCA AAA CTT GAG G-3’
IL-1β	Forward	5'-CTT TTC GTG AAT GAG CAG ACA G-3’
	Reverse	5'-TCA GCT TCA ATG AAA GAC CTC A-3’
IL-18	Forward	5'-GAA GAC TCT TGC GTC AAC TTC A-3’
	Reverse	5'-CTG ATT CCA GGT CTC CAT TTT C -3’

Supplementary Table 2. List of antibodies for immunohistochemistry and immunocytochemistry

Antibody name	Company	Dilution rate
TLR4	Novos Biological	1:200
NF-kB	Cell signaling	1:500
NLRP3	Abcam	1:200
ASC	Santa cruz Biotechnology	1:200
ICAM-1	Santa cruz Biotechnology	1:250
VCAM-1	Abcam	1:250
ET-1	Abcam	1:200

Supplementary Figure

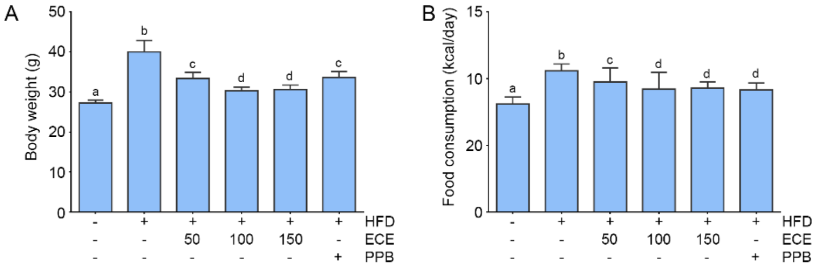

Figure S1. The reducing effects of ECE and PPB on body weight and food consuption in HFD -fed mice. (**A**) The graph of body weight in the HFD-fed mice. **(B)** The graph of food consumption in the HFD-fed mice. Data represent the means ± SD. Means identified to a different letter indicate significant differences between groups. ECE, extract of *Ecklonia cava*; HFD, high-fat diet; PPB, pyrogallol-phloroglucinol-6,6-bieckol; SD, standard deviation.

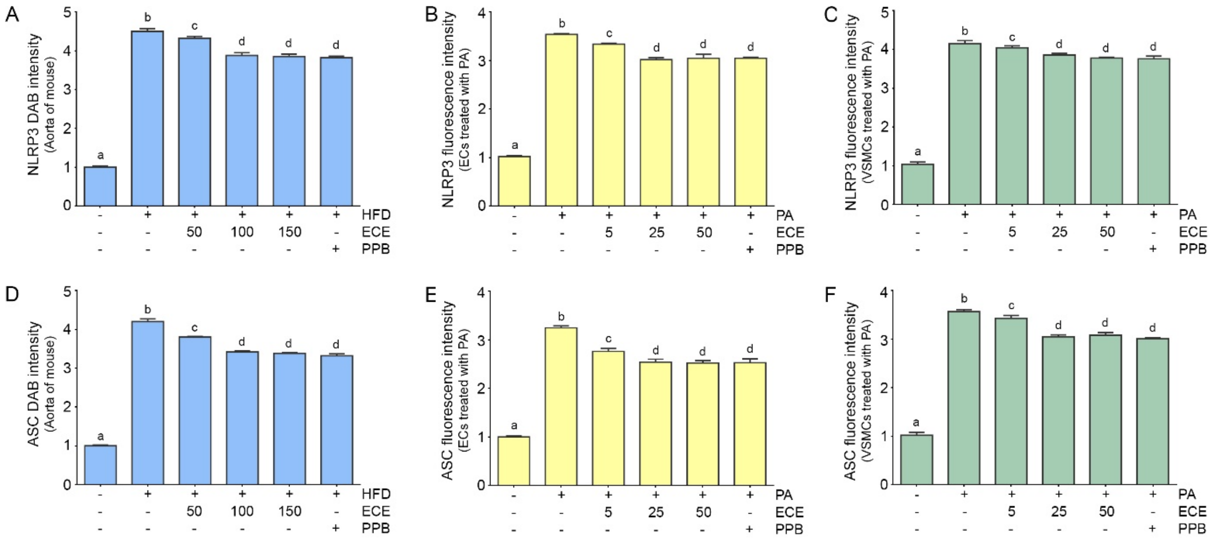

Figure S2. The reducing effects of ECE and PPB on expression of NLRP3 and ASC in the aorta and in the palmitate treated ECs and VSMCs. (**A**) The graph of NLRP3 protein expression level in the aorta. **(B, C)** In ECs (B) and VSMCs (C), the graph of NLRP3 protein levels. **(D)** The graph of ASC protein expression of ASC in the aorta was increased. **(E-F)** In EC (E) and SMC (F), the graph of ASC protein levels. Addition of ECE and PPB decreased the ASC expression levels Data represent the means ± SD. Means identified to a different letter indicate significant differences between groups. ASC, apoptosis-associated speck-like protein; DAB, 3, 3 -diaminobenzidine; ECs, endothelial cells; ECE, extract of *Ecklonia cava*; NLRP3, NOD-like receptor pyrin domain-containing protein 3; HFD, high-fat diet; PA, palmitate acid; PPB, pyrogallol-phloroglucinol-6,6-bieckol; SD, standard deviation; VSMCs, vascular smooth muscle cells.
